# Genome-wide transcriptome analysis of the orphan crop tef (*Eragrostis tef* (Zucc.) Trotter) under long-term low calcium stress

**DOI:** 10.1038/s41598-022-23844-z

**Published:** 2022-11-15

**Authors:** Ayalew Ligaba-Osena, Mohammad Salehin, Muhammad Numan, Xuegeng Wang, Sang-Chul Choi, Dereje Jima, Louis-Marie Bobay, Wanli Guo

**Affiliations:** 1 Laboratory of Plant Molecular Biology and Biotechnology, Department of Biology, University of North Carolina Greensboro, Greensboro, NC USA; 2 Laboratory of Environmental Epigenetics, Department of Biology, University of North Carolina Greensboro, Greensboro, NC USA; 3 Institute of Modern Aquaculture Science and Engineering, College of Life Sciences, South China Normal University, Guangzhou, 510631 People’s Republic of China; 4 Center of Human Health and the Environment (CHHE) and Bioinformatics Research Center (BRC), North Carolina State University, Raleigh, NC 27606 USA; 5 Laboratory of Microbial Genomics and Evolution, Department of Biology, University of North Carolina Greensboro, Greensboro, NC USA; 6 Department of Biotechnology, College of Life Sciences and Medicine, Zhejiang Sci-Tech University, Hangzhou, People’s Republic of China

**Keywords:** Physiology, Plant sciences

## Abstract

Calcium (Ca^2+^) is one of the essential mineral nutrients for plant growth and development. However, the effects of long-term Ca^2+^ deficiency in orphan crops such as Tef [(*Eragrostis tef*) (Zucc.) Trotter], which accumulate high levels of Ca in the grains, remained unknown. Tef is a staple crop for nearly 70 million people in East Africa, particularly in Ethiopia and Eritrea. It is one of the most nutrient-dense grains, and is also more resistant to marginal soils and climatic conditions than main cereals like corn, wheat, and rice. In this study, tef plants were grown in a hydroponic solution containing optimum (1 mM) or low (0.01 mM) Ca^2+^, and plant growth parameters and whole-genome transcriptome were analyzed. Ca^+2^-deficient plants exhibited leaf necrosis, leaf curling, and growth stunting symptoms. Ca^2+^ deficiency significantly decreased root and shoot Ca, potassium (K), and copper content in both root and shoots. At the same time, it greatly increased root iron (Fe) content, suggesting the role of Ca^2+^ in the uptake and/or translocation of these minerals. Transcriptomic analysis using RNA-seq revealed that members of Ca^2+^ channels, including the cyclic nucleotide-gated channels and glutamate receptor-like channels, Ca^2+^-transporters, Ca^2+^-binding proteins and Ca^2+^-dependent protein kinases were differentially regulated by Ca^+2^ treatment. Moreover, several Fe/metal transporters, including members of vacuolar Fe transporters, yellow stripe-like, natural resistance-associated macrophage protein, and oligo-peptide transporters, were differentially regulated between shoot and root in response to Ca^2+^ treatment. Taken together, our findings suggest that Ca^2+^ deficiency affects plant growth and mineral accumulation by regulating the transcriptomes of several transporters and signaling genes.

## Introduction

Calcium (Ca^2+^) is an essential plant nutrient with two crucial roles. It is a structural component of the cell wall and membranes^1^ where it binds to an acidic group of the membrane lipids and crosslinks pectin^2^. Ca^+2^ also acts as a ubiquitous second messenger in environmental stress signaling^1,3^. Several environmental stimuli lead to the release of stored Ca^2+^, which binds to calmodulin. The Ca^2+^-calmodulin complex recruits several kinases and phosphatases that regulate transcription-dependent cellular processes^4^. Plant cells also possess other Ca^2+^-regulated proteins, including Ca^+2^ dependent protein kinase (CDPKs), calcineurin B-like (CBL) proteins, CBL interacting protein kinases (CIPKs), CDPK-related kinases (CRKs), etc., which have been well characterized^5,6^.

In plants, Ca^2+^ is taken up from the soil solution by roots and translocated to the shoot via the xylem^4^. Ca^+2^ enters plant cells through Ca^2+^ channels^4^. Plant glutamate receptor-like (GLR) channels, which are analogs to the mammalian ionotropic glutamate receptors (iGluRs), are implicated in Ca^+2^ sensing, signaling, and long-distance root to shoot Ca^2+^ wave propagation during wounding^7,8^. Several members of the AtGLRs have been characterized^9,10^. The role of AtGLR3.1 in C^2+^ sensing in Arabidopsis shoot has been reported^11^. Another class of Ca^2+^ channels known as cyclic nucleotide-gated channels (CNGCs) has also been implicated in Ca^2+^ transport^12,13^. Cellular Ca^2+^ concentration is tightly regulated to allow signaling role, control Ca^2+^ translocation to the shoot and prevent cation toxicity^4^. Antiporters, uniporters and Ca^2+^-ATPases localized in all major plant cellular membranes and rapidly expel Ca^2+^ from the cytosol. The Arabidopsis genome encodes five different Ca^2+^ efflux systems, called autoinhibited Ca^2+^-ATPases (ACAs), ER-type Ca^2+^ATPases (ECAs), and P1-ATPases (HMA1), the mitochondrial calcium uniporter complex (MCUC) and Ca^2+^exchangers (CAX)^14,15^. Similarly, Ca^2+^/Cation-exchangers have also been reported to play a role in cation homeostasis in plants^16,17^.

Ca^2+^ deficiency is not commonly observed in most soils due to its relative abundance and regular liming practice^1,18^. However, developing tissues may experience Ca^2+^ deficiency due to lack of Ca^2+^ remobilization from older tissues, under conditions of low transpiration, in sandy soils untreated with lime (CaCO_3_ or Ca (OH)_2_), and inhibition of Ca^2+^ uptake due to competition from other cations^1,18^. Plants exposed to Ca^2+^ deficiency may experience necrosis of the meristems, shoot and root growth stunting^4^ and leaf carling. Ca^2+^-deficiency increases the level of reactive oxygen species, causing oxidative stress in high Ca-demanding plant species such as cabbage^19,20^ and broccoli^21^, Ca^+2^ treatment has been shown to enhance the antioxidant system and decrease oxidative stress in plants grown under high temperature^22^, drought^23^, salinity^24^ and heavy metals^25^.

Calcium-sensing networks, including the calcineurin B-like proteins (CBL)-CBL-interacting protein kinase (CIPK), has been implicated in plant abiotic stresses, including K^+^-deficiency^26^. The CBL1/9—CIPK23 has been implicated in the regulation of K^+^^27^ and Fe homeostasis (41). Similarly, CBL1/CBL9-CIPK11 interaction has been involved in regulating the bHLH transcription factor FIT, a crucial regulator of Arabidopsis Fe acquisition^28^. Fe deprivation has been linked to alteration of Ca^2+^ signatures evoked by exogenous ATP^29^, suggesting crosstalk between Ca^2+^ and Fe homeostasis. However, detailed studies on the interconnection between Ca^2+^ and Fe nutrition are missing. Recently, studies of factors modulating Ca^2+^-iron homeostasis are emerging^29,30^.

Tef is an important orphan crop in the Horn of Africa^31,32^ and some other countries including the United Sates where it is grown primarily for animal feed^33^. Recently, tef has been getting increased attention in many laboratories due to its health benefits and potential roles in sustainable agriculture^32,34^. Its mineral nutrient composition has been studied^35^. Tef is one of the orphan crops reported to have higher mineral contents; mainly, the Ca^2+^ content of tef is higher than other crops such as sorghum, barley, wheat, and rice^36^. The Ca^2+^ content in the plant varies from 0.1 to 5% of the shoot dry weight^4^ and the average seed Ca^2+^ content may vary between 981 and 1811 mg/Kg in tef as compared to 33 mg/Kg in maize, 153 mg/Kg in rice and 533 mg/Kg in wheat^35^.

Similarly, in millets such as finger millet, the Ca^2+^ contents were higher than in other major crops such as wheat, rice, and maize^37^. Low Ca^2+^ consumption in the diet has been related to several disorders in humans, many of which can have significant health repercussions over time. Because most staple food grains are low in Ca^2+^, tef, which is an orphan crop with high Ca^2+^ content, has enormous promise as a nutritional security crop. However, orphan crops’ physiological and transcriptomic response to Ca^2+^ nutrition has not been studied before.

This study exposed tef plants to long-term Ca^2+^ deficiency and analyzed the physiological and transcriptomic responses. Our findings revealed that prolonged low Ca^2+^ treatment decreased shoot and root biomass, Ca, K, and copper (Cu) content while significantly suppressing Fe translocation from the roots to the shoot. Transcriptomic analysis using RNA-seq revealed that some members of Ca^2+^ transporters, including CNGC, GLR and CaUP, signaling genes such as CBP and CDPK and genes implicated in Fe homeostasis such as YSL, OPT, VIT and NRAMP are differentially regulated by Ca^2+^ treatment or between roots and shoots.

## Results

### Low Ca^2+^ treatment inhibits plants growth

**Figure 1:**
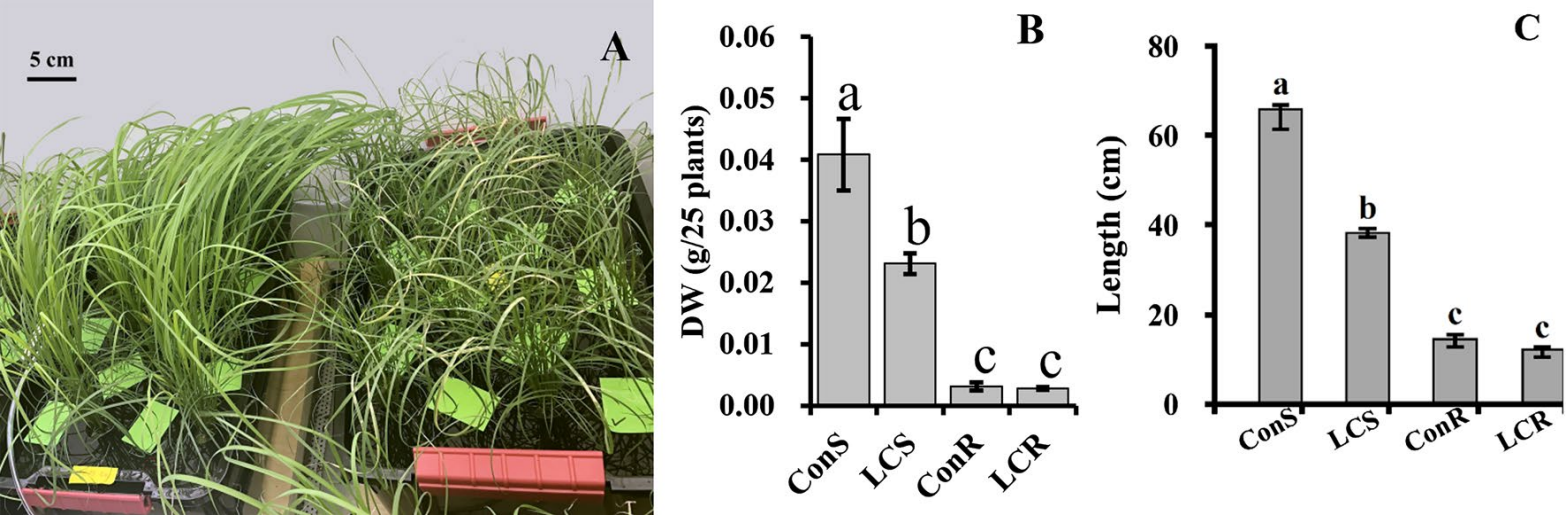
The phenotypic response of tef (*Eragrostis tef)* plants to long-term Ca treatment. (**A**) Phenotypes of tef plants treated with low (10 µM) or optimum or control (1 mM) calcium in modified Hogland’s hydroponics media for four weeks. The picture was taken using an Apple iPhone camera by AL-O. (**B**) Root and shoot biomass, and root length of tef plants grown in Hoagland solution containing 10 µM or 1 mM Ca. LCS, low calcium grown shoots; ConS, control/optimum Ca grown shoots. LCR, low calcium grown roots; ConR, optimum calcium treated roots. (**C**) Shoot and root length of tef plants grown under control (ConS and ConR) and low Ca (LCS and LCS) conditions. Bars represent mean and SD of at least 4 independent biological replicates, and different letters indicate statistically significant difference (*p* < 0.05).

To understand the effect of long-term Ca^2+^ deficiency in tef, plants were grown in a modified Hoagland’s hydroponic solution supplemented with predetermined low (0.01 mM) or optimum (1 mM) Ca^2+^ for one month, and the plants were analyzed. As shown in Fig. 1, plants grown in low Ca^2+^ exhibited typical Ca^2+^ deficiency symptoms, including leaf tip necrosis and growth stunting, compared to plants grown at optimum Ca^2+^ level (Fig. 1A). Plants were then dried at 65 °C overnight, and root and shoot dry matters were determined. As shown in Fig. 1B, Ca^2+^ deficiency decreases shoot and root biomass by 45% and 35%, respectively. The results also showed that prolonged low Ca^2+^ treatment decreased shoot and root elongation and plant height significantly from 66 to 38 cm, while root length was affected only slightly from about 15 cm to 12 cm (Fig. 1C).

### Ca^2+^ deficiency disturbs minerals homeostasis

**Figure 2:**
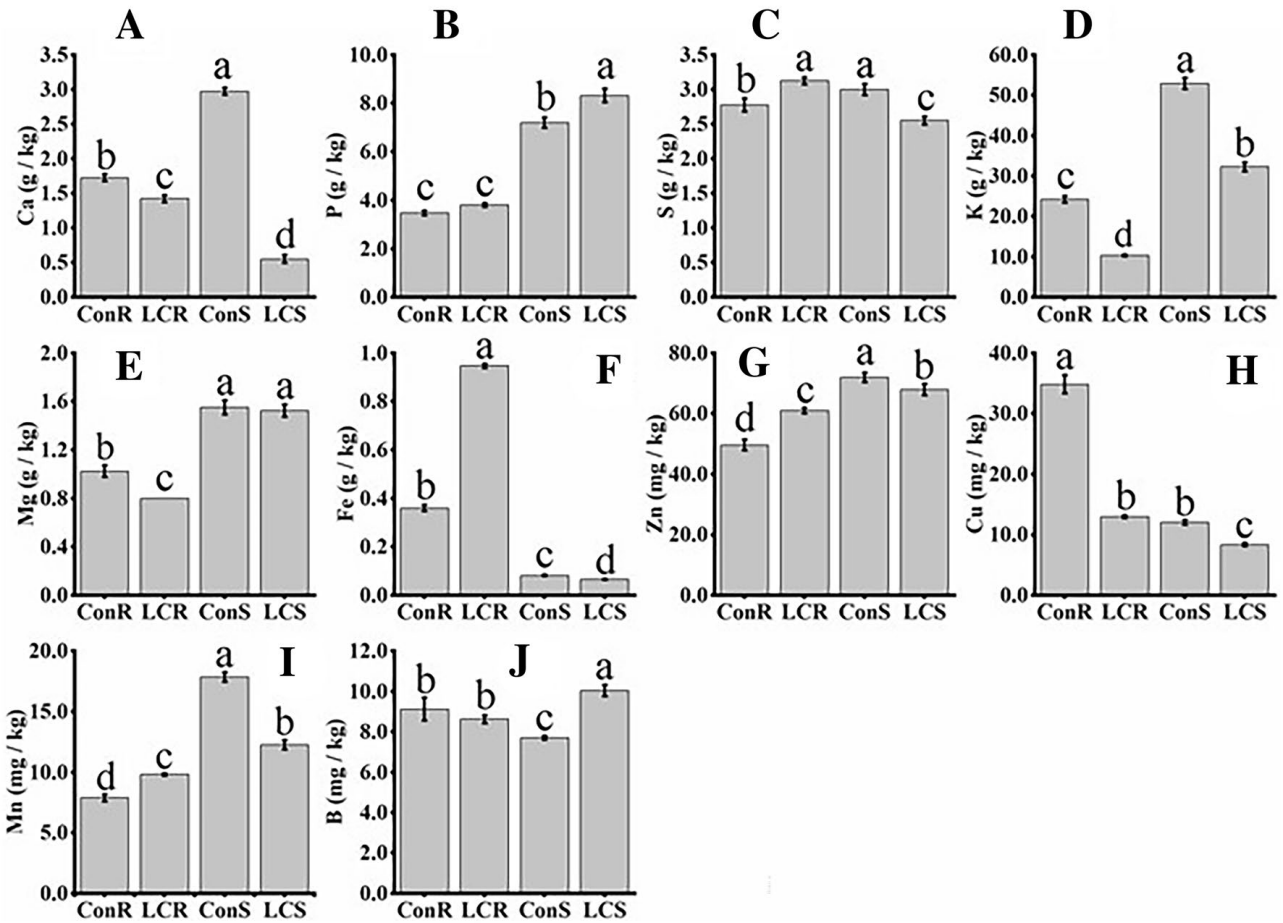
Mineral content profiling of tef plants grown under low or optimum Ca conditions. Shoot and root Ca (**A**), P (**B**), S (**C**), K (**D**), Mg (**E**), Fe (**F**), Zn (**G**), Cu (**H**), Mn (**I**) and B (**J**) were analyzed using ICP-OES as described in the “Materials and methods” section. Bars represent mean and SD of at least 4 independent biological replicates, and different letters indicate statistically significant difference (*p* < 0.05).

Fourteen-day-old seedlings were cultured in hydroponic solutions supplemented with 0.01 mM or 1 mM Ca^+2^ for one month. Mineral content from the dried shoot and root samples was determined using an inductively coupled plasma-optical emission spectrometer (ICP-OES). Plants grown in low Ca^2+^ media accumulated 0.5 g/Kg Ca^+2^ in the shoot (LCS) as compared to plants grown at optimum Ca^+2^ level (ConS), which accumulated 3 g/Kg Ca^+2^ (Fig. 2A). However, there was no statistically significant difference in root Ca^2+^ content. Similarly, there was no significant difference in root P content in plants grown at low (LCR) and optimum Ca^+2^ (ConR). At the same time, there was a small but statistically significant difference between LCS and ConR (Fig. 2B). S contents in root were slightly higher in plants grown at low Ca^2+^ while shoot S content was significantly lower in LCS than LCS (Fig. 2C). Ca^+2^ deficiency significantly suppressed root and shoot K content (Fig. 2D). In roots, K content decreased from 24.2 g/Kg in control plants to 10.3 g/Kg in Ca^2+^ deficient plants. Similarly, shoot K content decreased from 52.9 g/Kg in control plants to 32.2 g/Kg in Ca^+2^ deficient plants, suggesting the role of Ca^+2^ in K^+^ uptake and homeostasis. Ca^+2^ deficiency also decreased Mg content in the roots, but it did not affect shoot Mg content (Fig. 2E). Interestingly, Fe content in LCR was 2.5-fold higher than ConR, 0.95 g/Kg and 0.36 g/Kg, respectively (Fig. 2F). However, Fe content in LCS (0.06 g/Kg) was slightly lower than in control plants (0.08 g/Kg), suggesting that Ca^+2^ deficiency impairs root to shoot translocation of Fe. The Zn content was slightly higher in LCR as compared to ConR, while in shoots, Zn content was slightly lower in LCS than ConS (Fig. 2G). We also observed that Cu accumulation was significantly affected by Ca^+2^ treatment. Ca^+2^ deficiency decreased root Cu content by three-folds (from 0.04 g/kg in ConR to 0.013 g/Kg in LCS), while in shoots, a small but significant decrease in Cu content was observed (0.01 g/Kg in ConS to 0.01 g/kg in LCS) (Fig. 2H). Both zinc (Zn) and copper (Cu) contents were lower in shoots than in roots. The manganese (Mn) content in the roots was slightly higher in LCR than ConR, while Mn content was significantly lower in LCS as compared to ConS (Fig. 2I). B content in roots was not affected by Ca^+2^ treatment, whereas shoot B content was significantly higher in LCS than ConS (Fig. 2J). Taken together, Ca^+^^2^ deficiency markedly affects mineral homeostasis, primarily cations.

### Characteristics of RNA sequencing data

Since Ca^2+^ deficiency-induced visual symptoms in tef plants, including growth stunting and leaf necrosis in tef (Fig. 1A), we aimed at analyzing genome-wide transcriptomic changes in response to Ca^2+^ treatment. Total RNA was isolated from control and low Ca^2+^-grown shoots and roots (ConR, ConS, LCR, and LCS). Complementary DNA libraries were generated from four independent biological replicates and sequenced using the paired-end sequenced using Illumina Hiseq 4000. A total of 476.5 million raw reads with an average of 30 million reads were obtained for each sample The total clean reads of the 16 libraries were aligned to the tef draft genome sequence^38^. On average 85.7% of the reads were uniquely aligned to the reference genome (Tables 1, S1).

**Table 1:** RNA sequencing profile of tef samples under two different Ca^2+^ levels in root and shoots.

Treatments	LCR	LCS	ConR	ConS
Number of input reads	22,769,930	25,179,862.3	36,184,172.5	34,989,385.3
Number of uniquely mapped reads	18,361,838.8	21,930,270.8	28,709,324.8	31,726,862.3
Percentage of uniquely mapped reads	80.7	86.9	84.5	90.7
Number of reads mapped to multiple loci	1,956,523.75	1,285,027	2,371,076.75	1,791,236.5
Unmapped reads (too short) percent	8.45	5.115	6.555	5.1175

Samples were LCR (low calcium roots), LCS (low calcium shoots), ConR (control roots) and ConS (control shoots). Note that values are averages of reads from four biological replicates.

### Analysis of differentially expressed genes (DEGs)

The quality of sequence reads was assessed using fastqc application, and 15 poor-quality bases were trimmed from the 5′-end. The remaining good-quality reads were aligned to the tef reference genome using hisat2 aligner^39^. For each replicate, per-gene counts of uniquely mapped reads were calculated using the htseq-count script from the HTSeq^40^ python package. The count matrix was imported to R statistical computing environment for further analysis. Initially, genes that have no count in the replicate samples were discarded. The remaining count data were normalized and differentially expressed genes were identified using the DESeq2^41^ R package^42^.

**Figure 3:**
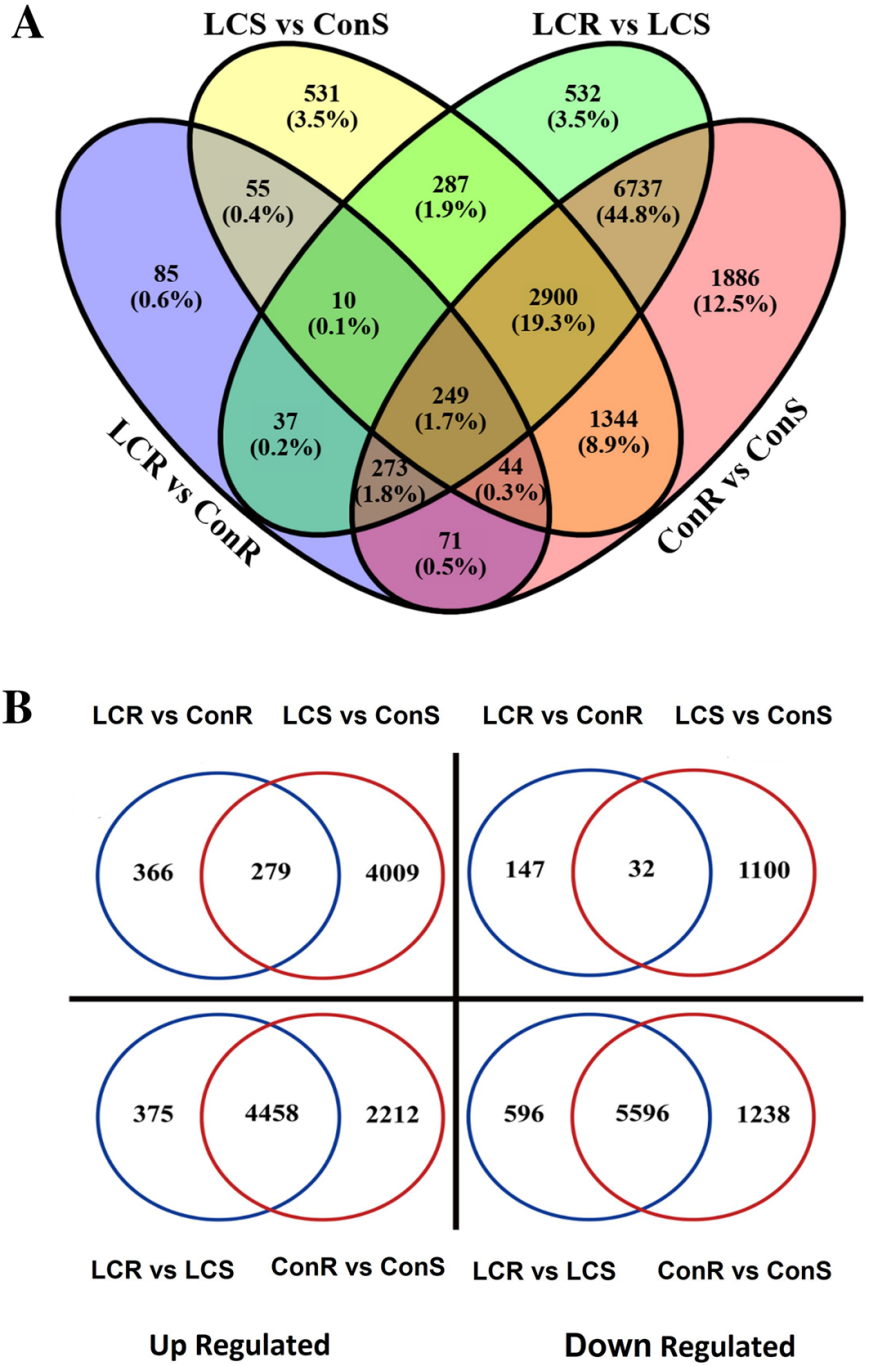
Transcriptomic response of tef in response to Ca^2+^ treatment. (**A**) Venn diagram illustrating the number of DEGs between the two Ca^2+^ treatment groups in root and shoot tissues, and (**B**) Venn diagram illustrating the number of differentially expressed genes between different treatment group. The treatment groups are LCR versus ConR, LCS versus ConS, LCR versus LCS and ConR versus ConS.

To determine the integrity of RNA samples, we performed principal component analysis (PCA). The PCA shows no mark variation among most RNA samples from the same Ca^+2^ treatment or tissue (Supplementary Fig. 1A,B). Our analysis identified many DEGs in response to Ca^+2^ treatment suggesting the crucial role of Ca^+2^ in plant physiological processes, including overall plant performance (Fig. 1) and mineral homeostasis (Fig. 2). The Venn diagram in Fig. 3A summarizes the pairwise comparisons of different groups (LCR, LCS, ConR, and ConS) based on the number of DEGs regulated in roots and shoots of tef plants grown under low and optimum Ca^+2^ conditions. As compared to ConR, 824 DEGs were detected in LCR, of which 85 are unique to LCR. A total of 5,420 DEGs out of 28,796 genes were detected in LCS as compared to ConS of which 531 DEGs were uniquely detected in LCS. Relative to LCS, 10,752 DEGs were seen in LCR of which 532 DEGs are unique to LCR. A total of 13,504 DEGs were detected in ConR as compared to LCR, of which 1,886 DEGs were uniquely detected in ConR. A total of 249 DEGs were common in all the pairwise comparisons (LCR vs. ConR, LCS vs. ConS, LCR vs. LCS, and ConR vs. ConS).

Figure 3B illustrates the transcriptomic response of tef plants grown in optimum and low Ca^2+^ conditions. A large number of genes (4.654) were upregulated in roots and shoots of low Ca^2+^ relative to control, of which 366 and 4009 genes were uniquely upregulated in LCR and LCS relative to ConR and ConS, respectively. A total of 279 genes were commonly upregulated in LCR and LCS. We also detected 1279 DEGs that were downregulated in LCR (179) and LCS (1132) compared to ConR and ConS, respectively. A total of 32 genes were commonly downregulated in LCR and LCS as compared to ConR and ConS, respectively. Ca^2+^ deficiency also induced significant transcriptomic changes between roots and shoots. A total of 7045 DEGs were upregulated in roots, of which 4833 DEGs were upregulated in LCR relative to LCS, 6670 DEGs were upregulated in ConR relative to ConS, and 4458 DEGs were commonly upregulated in both LCR and ConR. Similarly, 7430 DEGs were downregulated in roots, of which 6,192 DEGs were downregulated in LCR relative to LCS, 6834 DEGs were downregulated in ConR relative to ConS, and 5596 DEGs were commonly downregulated in both LCR and ConR relative to LCS and ConS, respectively. Taken together, the number of transcripts that were upregulated in response to Ca^2+^ deficiency in both roots and shoots were higher than those whose transcript abundance was downregulated. Moreover, the number of DEGs that were upregulated in ConR versus ConS were higher than those DEGs that were upregulated in LCR versus LCS (Fig. 3B).

### GO annotation of DEGs

**Figure 4:**
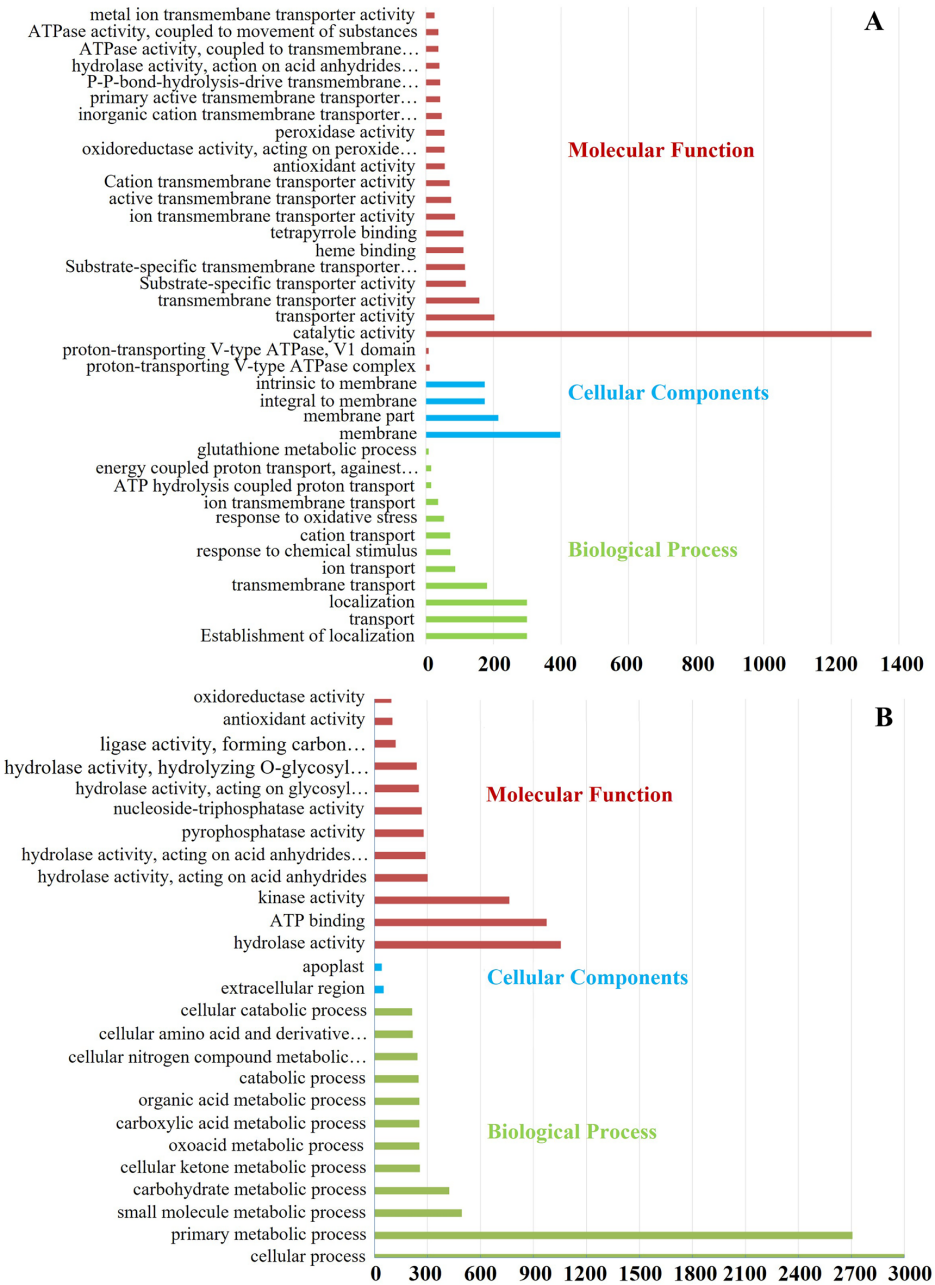
Gene Ontology (GO) enrichment terms of DEGs. Bars represent the number of genes enriched in LCR versus ConR (**A**), and the number of genes enriched in LCS versus LCS (**B**). GO annotation was performed as described in the “Materials and methods” section.

We performed Gene Ontology (GO) enrichment analysis using the AgriGo database to determine the potential function of DEGs identified in our analysis. In the GO analysis, the DEGs were grouped into molecular function, cellular components, and biological process categories (Fig. 4. Of these categories, DEGs with catalytic activity (> 1300 DEGs) were the most highly enriched group (Fig. 4A) for the LCR vs ConR comparison, followed by those predicted to have transport activity (200 DEGs). In the cellular component category, all the DEGs that were enriched are associated with membranes. In contrast, in the biological process category, about 300 DEGs are predicted to be involved in establishing localization, transport, and transmembrane transport. We also noted that DEGs with metal ion transporter activity, ATPase activity, primary active transporter activity, inorganic cation transporter activity, and substrate-specific transporter activity were on top of the list (Fig. 4A). When LCS versus ConS comparison was used for GO analysis, we observed that DEGs with hydrolase activity, ATP binding, and kinase activity were most enriched in the molecular function category. In contrast, in the biological process category, genes with cellular process followed by the primary metabolic process is the most enriched group (Fig. 4B).

### Calcium transporter genes are differentially regulated in response to Ca^2+^ treatment

**Figure 5:**
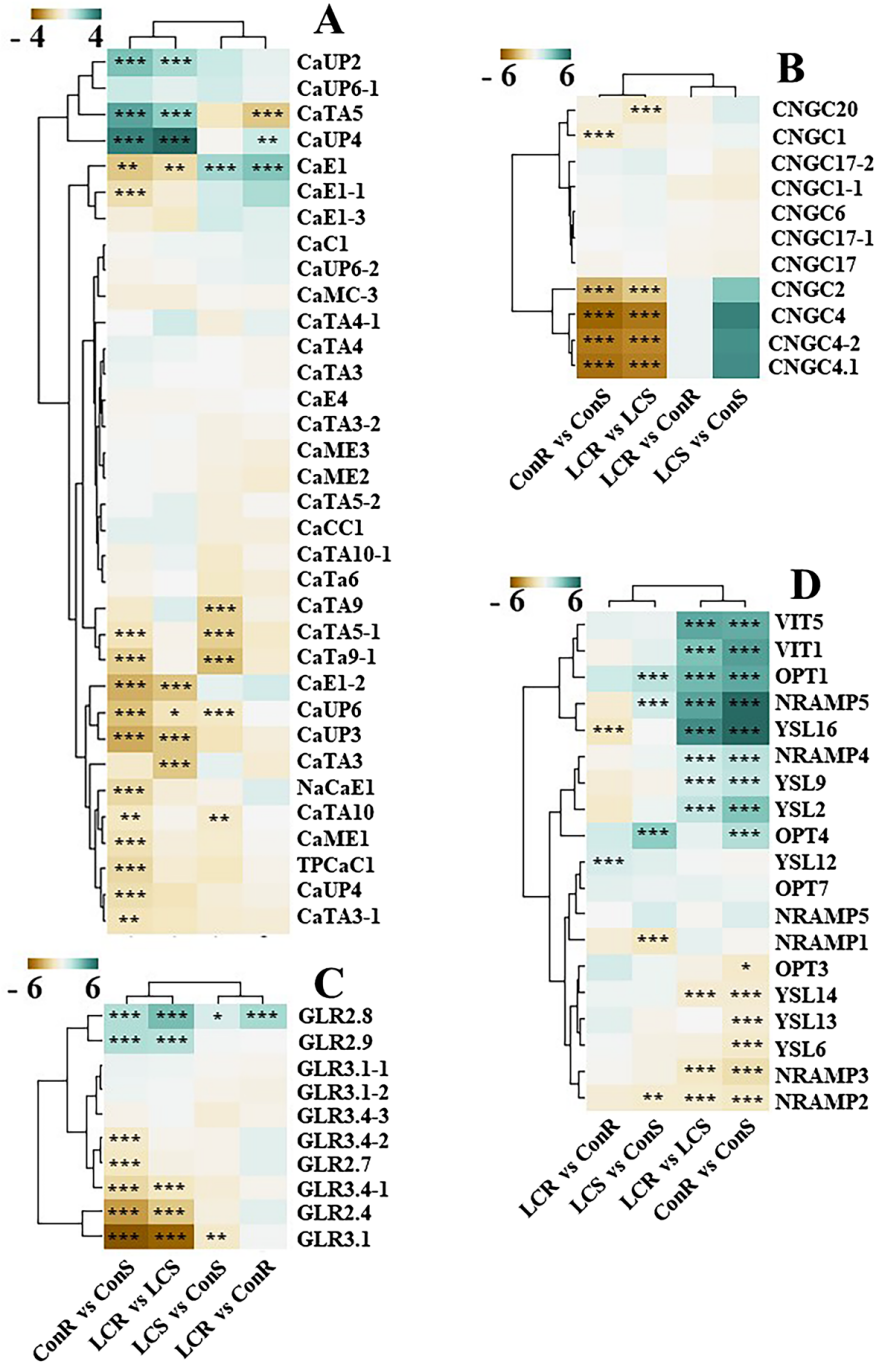
Heatmaps of differentially expressed genes. (**A**) Calcium transporters, (**B**) CNGCs, (**C**) GLRs and (**D**) Fe/metal transporters. Fe, iron, GLR, glutamate receptor; CNGC, cyclic nucleotide-gated channel, OPT, oligopeptide transporter; VIT, vacuolar iron transporter; NRAMP, metal transporter Nramp; YSL, yellow stripe-like; CaC1, calcium load-activated calcium channel; CaCC1, calcium permeable stress-gated cation channel 1; CaUP, calcium uniporter protein; CaMC, calcium-binding mitochondrial carrier protein; CaTA, calcium-transporting ATPase; CaE, cation/calcium exchanger; CaME, mitochondrial proton/calcium exchanger protein; NaCaE, sodium/calcium exchanger; TPCaC, two pore calcium channel protein. *p < 0.05; **p < 0.01; ***p < 0.001. (**A**) Et_4A_032234, CaC1; Et_3B_028475, CaCC1; Et_1A_007032, CaUP2; Et_4A_035795, CaUP4; Et_3B_029644, CaUP6; Et_10A_001249, CaUP6-1; Et_6A_048187, CaUP6-2; Et_1A_008615, CaUP3; Et_7B_055244, CaUP4; Et_2B_021219, CaMC-3; Et_4B_039870, CaTA10; Et_10B_003581, CaTA10-1; Et_4A_033710, CaTA3; Et_4B_037342, CaTA3-1; Et_4A_033168, CaTA3-2; Et_4A_034993, CaTA4; Et_9A_061403, CaTA4-1; Et_1A_007426, CaTA5; Et_8A_057406, CaTA5-1; Et_8A_057411, CaTA5-2; Et_7B_054771, CaTA3; Et_10A_001931, CaE1; Et_1A_006089, CaE1-1; Et_4A_035362, CaE1-2; Et_1B_010978, CaE1-3; Et_4A_035689, CaE4; Et_7A_050701, CaME1; Et_1B_014429, CaME2; Et_9A_061952, , CaME3; Et_9A_062174, CaTA6; Et_10A_001147, CaTA9; Et_6A_046490, CaTA9-1; Et_3A_023829, NaCaE1; Et_3A_024662, TPCaC1. (**B**) Et_1B_013097, OPT4; Et_1A_008454, YSL2; Et_1B_013348, YSL14; Et_2B_020454, VIT1; Et_2B_020954, Nramp3; Et_3B_028366, Nramp4; Et_3B_031448, YSL16; Et_4A_033828, OPT3; Et_4A_034655, Nramp2; Et_5A_041073, Nramp1; Et_5A_041086, Nramp5; Et_7A_051270, YSL6; Et_7A_052425, YSL9; Et_7B_055732, VIT5; Et_7A_053172, YSL13; Et_7B_055138, YSL12; Et_8A_058302, OPT1; Et_10B_003591, Nramp6; Et_2A_017928, OPT7. (**C**) Et_1A_006195, GLR2.7; Et_2A_016261, GLR2.4; Et_2A_016906, GLR3.4-1; Et_2A_018314, GLR2.9; Et_2A_018316, GLR2.8; Et_5B_043951, GLR3.4-2; Et_7A_052266, GLR3.1; Et_7B_054916, GLR3.1-1; Et_1A_008007, GLR3.1-2; Et_5B_045010, GLR3.4-3. (**D**) Et_1A_006672, CNGC20; Et_2A_016805, CNGC1; Et_3B_029538, CNGC4; Et_4B_038004, CNGC2; Et_4B_037294, CNGC4-1; Et_9B_064738, CNGC4-2; Et_1A_006188, CNGC17; Et_1A_007771, CNGC1-1; Et_2A_015772, CNGC17-1; Et_2A_017586, CNGC17-20; Et_7B_054569, CNGC6. The graphs were generated using R Software^42^(URL https://www.R-project.org/:).

Several families of genes have been implicated in Ca^2+^ transport in plants. This study identified transcriptomic changes in some families of Ca^2+^ transporters, including GLRs, CNGCs, Ca^2+^ uniporters, cations/Ca^2+^ exchangers, and Ca^2+^-transporting ATPases. Our analysis identified Ca^2+^ transporters including Ca^2+^ uniporters, cation/Ca^2+^ exchangers and Ca^2+^ transporting ATPase (Fig. 5A) etc. Expression of putative Ca^2+^ uniporters *CaUP2* and *CaUP4* were higher in roots than shoots in both low and optimum Ca^2+^ conditions. Transcripts of several transporters, including *CaE1-1, CaTA5-1, CaTA9-1, CaUP6, CaUP3, NaCaE1* were more abundant in ConS relative to ConR while transcripts of *CaE1-2, CaUP3, CaTA3,* and *CaUP4* were more abundant in LCR than LCS. Transcripts of CaE1 was more abundant in LCS than ConS, while *CaTA9, CaTA5-1, CaTA9-1, CaUP6,* and *CaTA10* were more abundant in ConS than LCS. *CaE1, CaE1-1,* and *CaUP4* are more abundant in LCR than ConR, whereas CaTA5 is more abundant in ConR than LCR. Transcripts of several members of Ca^2+^ transporting proteins are not markedly affected by Ca^2+^ treatment and between roots and shoots (Fig. 5A).

In response to Ca^2+^ treatment, several Ca^2+^- binding, Ca^2+^ signaling, and other transporters, including Ca^2+^ transporters and Ca^2+^-gated channels, are differentially expressed in both roots and shoots. We analyzed the expression of the CNGCs, which are implicated in the transport of Ca^2+^ and other cations (Fig. 5B). Transcripts of *CNGC2, CNGC4, CNGC4.1,* and *CNGC4.2* were more abundant in shoots than roots under low and optimum Ca^2+^ conditions (Fig. 5B). The transcripts of these genes were more abundant in LCS than ConS, although not statistically significant. Expression of *CNGC1* and *CNGC20* are significantly downregulated in ConR and LCR as compared to ConS and LCS, respectively. There was no difference in the expression of CNGCs between LCR and ConS.

Figure 5 also shows that several putative tef glutamate receptors such as *GLR3.1, GLR2.8, GLR4-1, and GLR3.4-1* are differentially expressed in response to Ca^2+^ deficiency, as shown in Fig. 5C. In ConS, transcripts of *GLR2.4, GLR2.7, GLR3.1, GLR3.4,* and *GLR3.4-1* were more abundant than ConR, while *GLR2.4, GLR3.1,* and *GLR3.4-1* were more abundant in LCS than LCR. However, the transcripts of *GLR2.8* were more abundant in LCR than ConR.

### Low calcium regulates the expression of Fe and metal transporter genes

Our analysis also revealed that several members of the putative iron transporters are differentially regulated in response to Ca^2+^ treatment and between tissue types (Fig. 5D). Transcripts of several tef homologs of *Arabidopsis* and rice iron transporter, including *VIT5, VIT1, OPT1, NRMAP5, YSL16, NRAMP4, YSL9,* and *YSL2* were more abundant in LCS and ConS than LCR and ConR, respectively. *YSL2* is a critical enzyme in long-distance Fe transport through the xylem. The VITs transport Fe across the vacuolar membrane, and Nramps and OPTs are involved in Fe other metal transport. The expression of *YSL14, NRAMP2,* and *NRAMP3* was downregulated under low Ca^2+^ conditions in both roots and shoots. Transcripts of YSL13 and YSL6 were more abundant in ConR than LCR. Transcripts of *OPT1, OPT4,* and *NRAMP5* were upregulated in LCR relative to LCS, while *NRAMP1* and *NRAMP2* were more abundant in LCS than LCR. Transcripts of *YSL16* is upregulated in ConS than ConR, while YSL12 is upregulated in ConR than ConS (Fig. 5B).

### Calcium signaling genes are differentially regulated by long-term Ca^2+^ treatment

**Figure 6:**
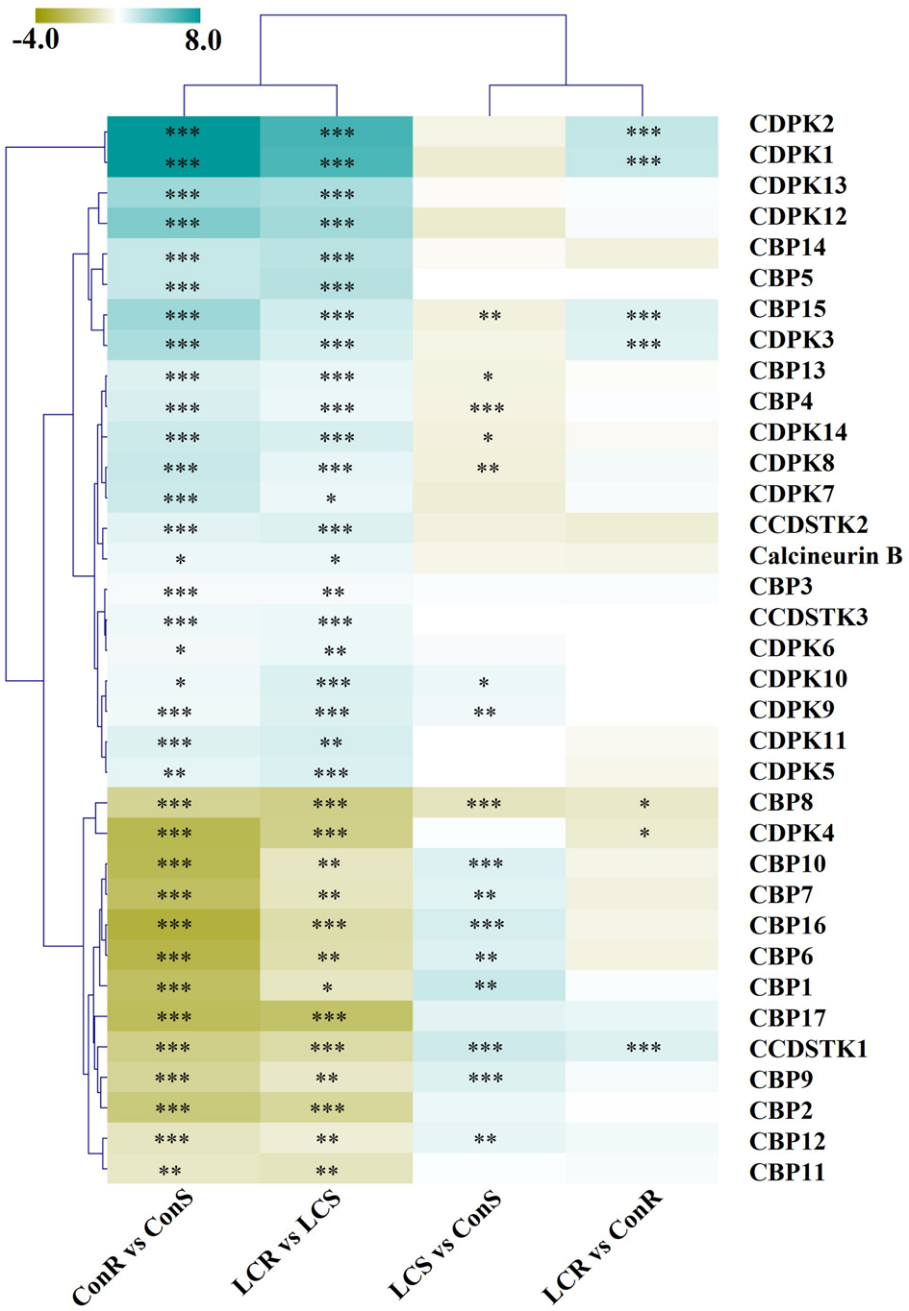
Heatmap of differentially expressed Ca^2+^-signaling genes. Several families of Ca^2+^-signaling genes are differentially regulated between the Ca treatment and root and shoot tissues. CCDSTK, calcium/calmodulin-dependent_serine/threonine-protein_kinase; CBP, calcium-binding_protein; CDPK, calcium-dependent_protein_kinase. Et_8B_060076, Calcineurin B; Et_4B_036878 CCDSTK1; Et_5A_041770, CCDSTK2; Et_5B_044444, CCDSTK3; Et_1B_010286, CBP1; Et_1B_014273, CBP2; Et_9A_062108, CDPK1; Et_9B_064669, CDPK2; Et_4A_033992, CDPK3; Et_7A_052314, CDPK4; Et_7B_054972, CDPK5; Et_2A_018014, CDPK6; Et_5A_040958, CDPK7; Et_5B_043654, CDPK8; Et_10A_000985, CDPK9; Et_10B_003165, CDPK10; Et_1A_008319, CDPK11; Et_4A_032792, CDPK12; Et_4B_036955, CDPK13; Et_5B_045743, CDPK14; Et_9A_063197, CBP3;Et_5B_044570, CBP4; Et_3A_023309, CBP5; Et_9B_066197, CBP6; Et_2B_022523, CBP7; Et_2A_018508, CBP8; Et_6A_046999, CBP9; Et_6B_049755, CBP10; Et_3A_023424, CBP11; Et_3B_027891, CBP12; Et_8A_057679, CBP13; Et_10A_001142, CBP14; Et_10B_004199, CBP15; Et_2A_017214, CBP16; Et_2B_021205, CBP17. The graph was generated using R Software^42^(URL: https://www.R-project.org/).

Because Ca^2+^ plays a key role in various abiotic stress signaling, we analyzed whether transcripts of signaling genes respond to long-term Ca^2+^ deficiency. As shown in Fig. 6, many signaling genes, including CBP and CDPKs differentially regulated by Ca^2+^ treatment and between root and shoots. Transcripts of several genes, including *CDPK4*, *CBP6*, *CBP16,* and *CBP17,* are more abundant in shoots than roots under both Ca^2+^ levels. At the same time, transcripts of several CDPK and CBP, including *CDPK1, CDPK2, CDPK12, CDPK3,* and *CBP15,* were more abundant in the roots of both control and low Ca^2+^ conditions. In roots, the expression of *CDPK1, CDPK2, CDPK3, CCDSTK1,* and *CBP15,* is upregulated by Ca^2+^ deficiency. Overall, our findings highlight the differential expression of some signaling genes in response to long-term Ca^2+^ deficiency.

### Validation of RNA-seq DEGs

**Figure 7:**
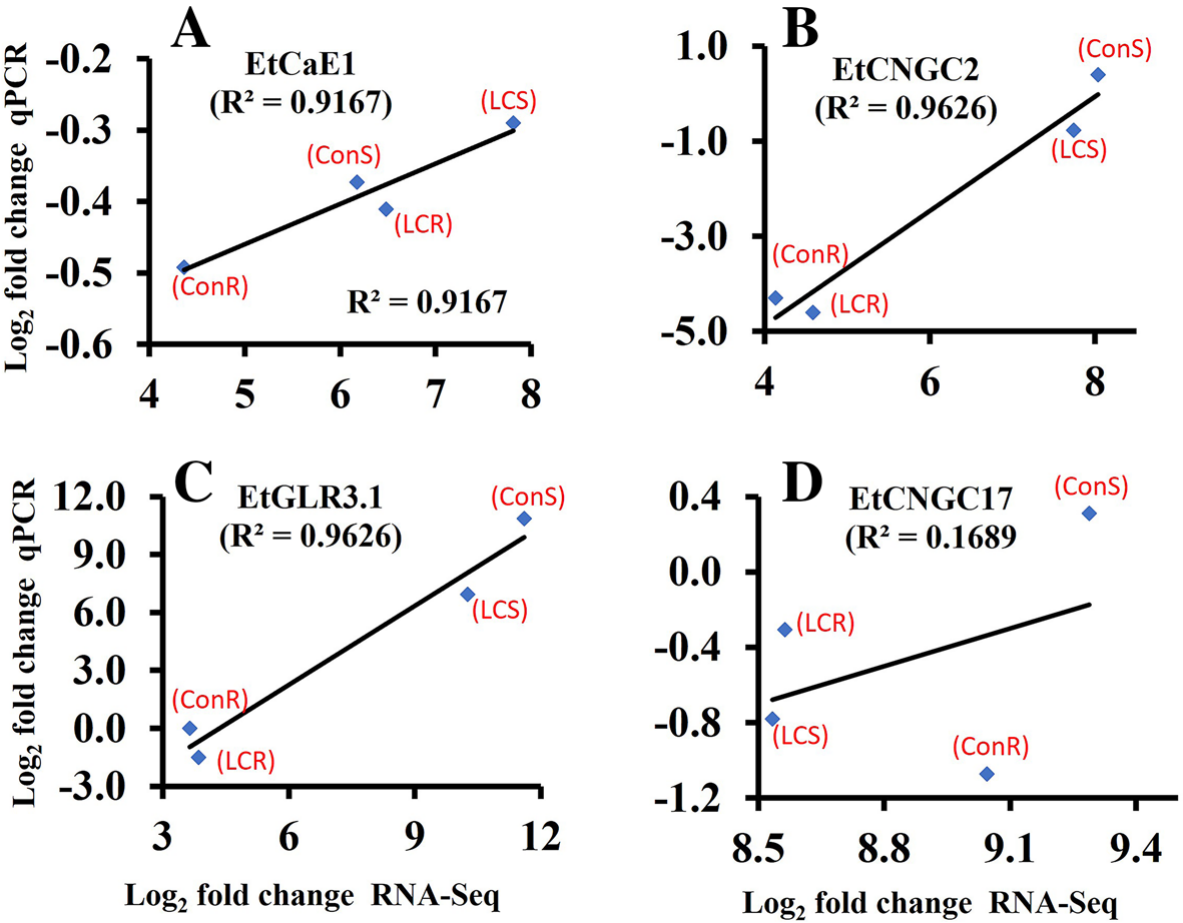
Validation of RNA-seq data by qPCR. Pearson correlation analysis of Log_2_ fold changes of DEGs based on RNA-seq and qPCR analysis. Data points and corresponding treatments (ConR, ConS, LCR and LCS) are shown.

To the RNA-seq data, we performed quantitative RT-PCR (qPCR) to analyze the expression of a few DEGs, including *CaE1, CGC2, GLR3.1,* and *CNGC17*. As shown in Fig. 7, we performed Pearson correlation of Log^2^ fold changes in gene expression by RNA-seq and qPCR analysis. A strong positive correlation was observed between the two assays for *CaE1* (R^2^ = 0.9167), *CNGC2* (R^2^ = 9626) and *CaE1* (R^2^ = 9626), while a positive but weak correlation was observed for CNGC17 (R^2^ = 0.1689). These findings validate the RNA-seq data.

## Discussion

The Chloridoideae subfamily of Poaceae, including tef and finger millet, accumulate higher levels of Ca^+2^ in grains than other cereals^43^. However, the effect of prolonged Ca^2+^ deficiency on their physiology and transcriptome remained unknown. In the present study, we examined the response of tef to prolonger Ca^2+^ deficiency based on agronomic, physiological, and transcriptome changes in plants grown in hydroponics solution under optimum and Ca^2+^ deficient conditions.

Tef plants grown under Ca^2+^ deficient conditions showed typical deficiency symptoms in shoots, including leaf curling, tip necrosis, and growth stunting (Fig. 1A), which is in line with previous reports^44^. We performed the elemental analysis of the shoots and roots to validate our phenotypic analysis. Our findings revealed that prolonged Ca^2+^ deficiency significantly decreased Ca^2+^, K, and Cu content in both the roots and the shoots, and S, Zn, and Mn in the shoots. Interestingly, Fe translocation from roots to shoots is significantly suppressed by Ca^2+^ deficiency. Root Fe content was 2.5-fold higher in roots than in shoots. These suggest that Ca^2+^ deficiency suppresses plant growth by perturbing homeostasis of essential minerals, including macro and micronutrients. Ca^2+^ deficiency is rare in nature but may happen when plants are grown in acidic soil^4^. Although there is no report on the effect of Ca^2+^ deficiency on plant Fe translocation, some studies have reported on the interaction between Ca^2+^ and Fe. A high level of Ca^2+^ has been reported to cause Fe chlorosis by suppressing Fe acquisition in field crops^45^ and decreased Fe uptake in dry bean plants^46^.

To understand the transcriptomic change in response to Ca^2+^ deficiency, we performed RNA-seq using the Illumina HiSeq platform. Our analysis revealed that many transcripts are differentially regulated between control and Ca^2+^ deficient conditions, and between roots and shoots of plants grown under both Ca^2+^ treatments (Figs. 3 and 5). Moreover, our GO analysis highlighted significant enrichment of several Ca^2+^ transporters, signaling genes, those genes with transport activities, ATPase activity, catalytic activities etc. (Fig. 5). Such transcriptomic changes are expected since Ca^2+^ is an integral part of plant cell walls, mineral homeostasis, and signaling^44^.

When plants undergo certain environmental stresses, Ca^2+^ signals are generated, which trigger a rapid rise in cytosolic Ca^+2^ levels^47^. Several protein families are implicated in plants’ Ca^2+^ transport processes, including Ca^2+^-permeable ion channels, Ca^2+^/H^+^ exchangers, and pumps (Ca^2+^-ATPases)^1^. Several Ca^2+^-permeable channels have been implicated in the uptake of Ca^2+^ from the soil solution to plant roots^48^. These include the GLRs, CNGCs, two-pore channels (TPCs), mechanosensitive-like channels (MSLs) etc.^49^. Ca^2+^ pumps are involved in maintaining homeostasis by controlling Ca^2+^ efflux from the cytosol to organelles and/or to the apoplast^50^ . In our dataset, we identified several DEGs including Ca^2+^ uniporters (CaUPs), cation/Ca^2+^ exchangers (CaE), Ca^2+^-transporting ATPases (CaTA) etc. (Fig. 5). Our findings revealed that that the *CaE1-1, CaTA5-1, CaTa9-1, CaE1-2, CaUP6, CaUP3, NaCaE1, CaTA10, CaME1, TPCaC1, CaUP4* and *CaTA3-1* were downregulated in ConR as compared to ConS (Fig. 5A). Whereas CaE1, CaE1-2, CaUP3, and CaTA3 were downregulated in LCR compared to LCS (Fig. 5A). When the LCS was compared to ConS, the *CaE1* was upregulated, and *CaTA9, CaTA5-1, CaTA9-1, CaUP6,* and *CaTA10* were downregulated. Similarly, when LCR was compared with ConR, the CaUP4 and CaE1 genes were upregulated, and *CaTA5* was downregulated (Fig. 5A). For example, *CaUP2, CaTA5,* and *CaUP4* were more highly expressed in ConR and LCR than ConS and LCS, respectively (Fig. 5A). It was previously reported that Ca^2+^ uniporters play an important role in stress signaling in Arabidopsis^51^. In another study, Ca^2+^ uniporter proteins were implicated in pollen tube germination^52^. As reported in previous studies, Ca^2+^ uniporters play an important role in Ca^2+^ signaling and homeostasis in Arabidopsis^51^ and homologs in mammals play role in Ca^2+^ uptake into the mitochondrial matrix and regulate calcium signaling, aerobic metabolism, and apoptosis^53^. None of these transporters have been isolated and characterized from tef before. However, several homologs have been characterized in other plant species such as Arabidopsis^54^, rice^55^ and apple (*Malus domestica*)^56^.

The CaCA superfamily has been reported to facilitate Ca^2+^ transport against its concentration gradient and enhance the influx of monovalent cations (K^+^, Na^+^, and H^+^)^57^. For example, overexpression of the apple cation/Ca^2+^ exchanger MdCCX1 in transgenic Arabidopsis and apple root cells has been shown to decrease Na^+^ accumulation and ROS levels and increase salt tolerance in transgenic cells^56^. In this study, Ca^2+^ deficiency decreased Ca and K content in roots. Therefore, upregulation of CaE1 in LCR and LCS could be a mechanism by which roots enhance K^+^ or Ca^2+^ acquisition from the external solution or facilitate their distribution between tissues. Transcripts of *CaTA9-1, CaTA5-1, CATA9,* and *CaUP6* are downregulated by Ca^2+^ deficiency in the shoots. The CaTA5 was the only gene among the transporters that was downregulated by Ca^2+^ deficiency in roots. CaTA5 is a homolog of the plasma membrane Ca^2+^-transporting ATPases (PMCAs), which are high-affinity Ca^2+^-pumps that export Ca^2+^ ions from the cytosol into the extracellular solution^58^. CaTA5 may be a functional Ca^2+^-pump, and its downregulation under Ca^2+^ deficiency could be a strategy to suppress the cytosolic Ca^2+^ export to the extracellular solution. The autoinhibited Ca^2+^-ATPase ACA13 has been implicated in successful pollination in the *Brassicaceae* by exporting pollen to compatible pollen tubes^59^.

This study also identified various Fe/metal transporters, including OPTs, VITs, NRAMPs, and YSLs. All these transporters proteins were differentially expressed in the roots and shoots of low Ca^2+^ and control plants. In Arabidopsis, *Oligopeptide Transporter 1(AtOPT1)* is a major nitrate/oligopeptide transporter family^60^. The Arabidopsis OPT3 is a phloem-specific iron transporter^61^ and its rice counterpart, OsOPT7, has been implicated in Fe loading into the phloem, the long-distance Fe transport, and loading to seeds^62,63^. OPT4 is involved in oligopeptide transport^64^. Furthermore, Arabidopsis *Oligopeptide Transporter 6* (*AtOPT6*) was reported to transport glutathione^65^ and a possible connection between prolonged Ca^2+^ deficiency, redox status, and increased iron transport is a likely scenario as suggested in a recent review^28^. In this study, transcript levels of several transporters such as *OPT1, EtNRAMP5*, *EtVIT5* and *EtYSL2* were upregulated by several folds (Fig. 5B) in the shoot during Ca^2+^ deficiency. Similarly, the *VIT1, YSL16, NRAMP4, YSL9* and *OPT4* were upregulated in both LCR vs. LCS and ConR vs. ConS. The transcripts of *OPT1, NRAMP5,* and *OPT4* were also upregulated, while that of *NRAMP1* and *NRAMP2* were downregulated in the LCR compared to ConR (Fig. 5D). The maize ZmYS1^66^ and rice OsYSL15^67^ import Fe^3+^-phytosiderophore (PS) across the root cell membrane, while the rice OsYSL2 is implicated in the long-distance transport of Fe associated with a PS precursor, nicotianamine (NA)^68^. The role of YSL2 in Fe uptake and distribution has been studied previously in maize^69^ and Arabidopsis^70,71^. The NRAMPs such as AtNRAMP3 and AtNRAMP4 function in transporting Fe out of vacuoles under Fe-limited conditions, while members of the VIT family mediate Fe transport into vacuoles^72^. For example, overexpression of the wheat VIT2 under the control of an endosperm-specific promoter increases Fe content in white flour fractions of wheat^73^. Some of these transporters are not specific to Fe but also transport other minerals, including Zn, Cd, Cu, and Mn^74^. Taken together, differential regulation of several mineral transporters in response to Ca nutrition suggests the involvement of Ca^2+^-signaling in the homeostasis of these minerals. The decrease in root to shoot Fe translocation in this study due to Ca^2+^ deficiency (Fig. 2F) suggests the involvement of Ca^2+^ signaling in Fe homeostasis. Ca^2+^-signaling has been implicated in the homeostasis of minerals such as K^27^. In this regard, Gratz et al.^75^ reported that CIPK11-dependent phosphorylation modulates the activity of FIT (a transcription factor that regulates Fe acquisition), promoting Fe uptake in Arabidopsis. Ca^2+^ promotes the interaction of C2-Domain Protein Enhanced Bending1(EHB1) and the root Fe-regulated transporter IRT1, inhibiting Fe acquisition in Arabidopsis^76^. However, further studies are needed to understand the crosstalk between Fe and Ca nutrition in Ca and Fe-rich cereals such as tef and finger millet.

Our study also revealed that several Ca^2+^-transporting channels were differentially expressed by Ca treatment. Our findings showed that transcripts of the CNGC, including *CNGC2, CNGC4, and CNGC4-2,* were more abundant in the shoots regardless of the Ca treatment (Fig. 5B). This may suggest that the CNCG may be involved in Ca^2+^ distributions in the shoots. The CNGC has lower ion selectivity permeating K^+^, Na^+^ and Ca^2+^ across the plasma membrane. The Arabidopsis genome consists of 20 members of the CNGC, and all the CNGC tested so far transport K^+^, while some members of the CNGC also translocate Ca^2+^^77. ^The *Arabidopsis* CNGC2 and CNGC4 are implicated in pathogen-induced Ca^2+^-signaling^78^. The CNGC2 and CNGC4 have also been implicated in plasma membrane external ATP-activated Ca^2+^ influx in pollen tubes of *Arabidopsis*^79^. As shown in Fig. 5C, *GLR2.8* and *GLR2.9* were significantly upregulated in ConR compared to ConS while *GLR3.4-2, GLR2.7, GLR3.4-1 GLR2.4* and *GLR3.1* were downregulated. Similarly, *GLR2.8* and *GLR2.9* were upregulated in LCR compared to LCS, while *GLR3.4-1, GLR2.4,* and *GLR3.1* were downregulated. In *Arabidopsis,* the GLRs such as GLR 3.1, GLR 3.2, and GLR 3.3 GLR 3.6 have been reported to mediate long-distance Ca^2+^ signaling during wound responses^10^. Heterologous expression of the rice OsGLR2.1 in mammalian cells resulted in Glu-triggered Ca^2+^ increase^30^. Similarly, in the basal land plant mosses (*Physcomitrella patens)*^9^, it has been shown that GLR1 encodes a non-selective ion channel that is permeable to Ca^2+^. Upregulation of *GLR2.8* and *GLR2.9* in roots compared to shoots in this study may suggest their involvement in Ca^2+^ uptake in the roots. Upregulation of *GLR2.8* in LCR compared to ConR may suggest its role in Ca^2+^ acquisition under Ca^2+^-deficient conditions. However, the GLRs which were upregulated in the shoots under both high and low Ca^2+^, such as *GLR3.4-1, GLR2.4,* and *GLR3.1,* may play a role in Ca^2+^ distribution in the shoots, which remains to be studied in the future.

In conclusion, we found that Ca^2+^ deficiency severely inhibits the growth of tef plants by disturbing the homeostasis of some minerals, including Ca, K, Fe, Mn, and Cu. Furthermore, our transcriptomic analysis revealed various genes, including those implicated in Ca^2+^-signaling, Ca^2+^- transporters, and Ca^2+^-permeable channels; Fe and metal transports which were differentially regulated by Ca^2+^ deficiency and/or between root and shoot tissues, suggesting their involvement in Ca^2+^ homeostasis. Heterologous expression and loss-of-function mutations studies of the DEGs identified in this study would elucidate their physiological functions in tef.

## Materials and methods

### Plant materials and growth condition:

We recently performed seed mineral profiling of diverse tef (*Eragrostis tef*) accessions obtained from the USDA-GRIN germplasm collection^43^. We used the accession with the highest seed Ca (1811 mg/kg) content in this study. Tef seeds were sterilized according to a method previously published with slight modifications^80^. Briefly, seeds were soaked in 70% ethanol for one minute, followed by soaking in 1% NaOCl solution containing 0.1% Tween-20 for 10 min with vortexing every 1 min. Subsequently, the seeds were washed with autoclaved high purity water (18.2 MΩ (megaohms)) five times. The seeds were then transferred to a petri-dish with a sterile wet filter paper disc. The plates were incubated in a growth chamber (Conviron, Winnipeg, Canada) for six days under 16 h/8 h day-night cycles at 28 °C. The seedlings were transferred to a modified Hoagland hydroponic solution^81^ in which the Ca(NO_3_)_2_ was replaced with NH_4_NO_3_ to investigate the effect of Ca^2+^. Plants were grown in the media containing either 1 mM Ca^2+^ (optimum or control) or 10 µM (low Ca or Ca-deficient). The nutrient solution was continuously aerated and was renewed every 4 days.

### Plant phenotyping/photography

Images were taken from the plants grown in a hydroponics system after ~ 28 days. Root and shoot dry weight biomass were measured at the end of the prolonged Ca^2+^ deficiency treatment. The measurements were taken for all replicates and mean values were calculated.

### Mineral element analysis

One-month-old tef plants grown in 1/4th-strength Hoagland’s hydroponics solution was analyzed. Roots and shoots were separated and rinsed three times with Milli-Q (18.2 MΩ) water. Before weight measurements, the tissues were oven dried at 65 °C overnight and ground to fine powder using a Waring Laboratory spice blender. Briefly, 500 mg of ground tissue was digested using concentrated HNO_3_ for 30 min in a microwave at 200 °C, and mineral content was analyzed using Inductively Coupled Plasma Optical Emission Spectroscopy (ICP-OES).

### Library construction and RNA sequencing

For RNA extraction, root and shoot samples were ground into powder under liquid nitrogen using a mortar and pestle. Total RNA was isolated using the GeneJET RNA purification kit following manufacturer’s procedure (Thermo Fisher Sci., Waltham, MA). The quality and quantity of the RNA were determined using a Nanodrop (Thermo Fisher Sci, Waltham, MA). mRNA-seq libraries were prepared using NEBNext® Ultra II RNA Library Prep Kit for Illumina (New England Biolab EB, Ipswich. MA) according to the manufacturer’s instructions. For each library, 500 ng total RNA was used. The mRNA-seq libraries were sequenced on an Illumina Hiseq platform using the 150 bp paired-end sequencing strategy.

### RNA-seq data analysis

Data analysis for RNA-Seq was performed by the Bioinformatics Core associated with the Center for Human Health and Environment at North Carolina State University. Quality control of read data was briefly evaluated with FastQC, and alignment trimming was performed using *sickle* with the options − q 12 and − l 15 (version 1.33^82^. Alignment was performed using *hisat2* short read aligner to *Tef* reference genome downloaded from CoGe Comparative Genomics website (V3.1, id59854)^38^. All default parameters were used except *–very-sensitive and –summary-file* options passed to the *hisat2* aligner^83^. The number of reads mapped to a genome feature was determined using *htseq-count* command line script from the *HTSeq* python package^84^ *(-s reverse -a 10 -t exon -I Parent -m union*) and count data was imported to the R statistical computing environment for further analysis^42^. Initially, genes that have no count in at least 4 of the replicate samples were excluded from the analysis, and the remaining 28,796 features were used for downstream analysis. Data normalization based on dispersion and differential analysis were conducted using the DESeq2 package^85^. We fitted a generalized linear model *(*~ *Treatment)* between the expression count. Finally, differential expression analysis was performed between ConR verus LCR, ConS versus LCS, Con R versus ConS, and LCR versus LCS, and differential expressions were identified by applying multiple testing correction using Benjamin-Hockberg procedure^86^.

### Quantitative reverse transcription PCR (qRT-PCR)

To validate the RNA-seq data for representative genes, quantitative real-time reverse transcriptase-polymerase chain reaction (qRT-PCR) was performed using a QuantStudio 3 real-time PCR system and the PowerUP™ SYBR Green pre-formulated 2 × master mix (Applied Biosystems, Foster City, CA). Total RNA was treated with DNase I (Invitrogen, Carlsbad, CA) to remove contaminating genomic DNA before first-strand cDNA synthesis. First-strand cDNA was synthesized using 5 µg of total RNA and the SuperScript® III First-Strand Synthesis System (Thermo Fisher, Waltham, MA) following the manufacturer’s instructions. First-strand cDNA was used in the real-time qRT-PCR reaction containing 2xSYBR Green Master Mix, 500 nM forward, and reverse gene-specific primers in a 20 uL reaction volume. Gene-specific sense and antisense primers 5′-tttggctcctgggactcctaac-3′ and 5′-ggccagattgcagtgtagagtttc-3′ for *GLR3.1*, 5′-tcatcttccatgctgccaaaggc-3′ and 5′-tctccagttgggccattgtgtc-3′ for *CNGC2*, 5′-gcgactcgaggtgtgaatgaagag-3′ and 5′-aatggaacccgccgaacaagac-3′ for *CNGC17,* and 5′-tgctgcaattccagagggcttg-3′ and 5′-tcacagttgtgcaacccaatgtc-3′ were used to amplify *CaE1*. The internal control, eukaryotic elongation factor *(EF1A)* was used as a housekeeping gene and amplified using sense 5′-catcaacatcgtggtcatcg and antisense 5′-gatgcctccaagcttgtaga-3′ primers. Gene expression in the untreated samples were used as a calibrator, and blank (without template) was used as a reference control. The qPCR parameters were as follows: 95^0^C for 30 s, 40 cycles of 95 °C for 10 s, 60 °C for 30 s, and a final incubation at 72 °C for 5 min followed by melting curve analysis. Relative expression levels based on fold changes were calculated using the ΔΔCT method^87^ available with the QuantStudio3 software (Applied Biosystems, Foster City, CA, USA) and in MS-Excel (Microsoft Corp, Seattle, WA).

### Gene annotation

Coding sequences (CDS) of *Eragrostis tef* were annotated by comparing it to the genome sequences of *Setaria italica* (GCF_000263155.2), *Oryza sativa*^88^, and *Sorghum bicolor* (GCF_000003195.3). The CDS of *E. tef* were compared to *S. italica*, *O. sativa,* and *S. bicolor* using Blastx. A CDS was considered significant with *e*-value < 0.00001. Significant hits were first annotated by comparison to *S. italica* (25,876 significant CDS), then to *O. sativa* (873 additional CDS) and *S. bicolor* (483 additional CDS). A total of 94.6% of the CDS of *E. tef* had a significant hit to a CDS of these three genomes, and the corresponding gene annotations were used.

### GO analysis

Go analysis was performed using the agriGO^89^. Signature genes from the RNAseq analysis were used as an INPUT, and significantly enriched pathways and/or families were selected based on molecular function, cellular structure, and biological process category.

### Data analysis

Treatments were replicated four times, and two independent experiments were conducted. Data were analyzed by the one-way ANOVA using the PROC GLM procedure in SAS^90^. After the significant F-tests, the Tukey’s multiple comparisons^92^ were used to separate the means (P ≤ 0.05). Values represent mean ± SD of six (Figs. 1B,C) and four (Fig. 2) biological replicates.

### Statement on ethics and approvals to use tef

Tef is primarily cultivated in East Africa and is becoming popular in several countries including South Africa, Australia, and the U.S. The tef accessions used in this study was obtained from Dr. John Cushman at the University of Nevada Reno. All methods are performed in accordance with relevant guidelines and regulations.

## Supplementary Information

Supplementary Information 1.

Supplementary Information 2.

## Data Availability

Sequence data files have been stored at https://www.ncbi.nlm.nih.gov/geo/query/acc.cgi?acc=GSE201043.
