# Supplementary figures and images for: Genome-wide transcriptome analysis of the orphan crop tef (Eragrostis tef (Zucc.) Trotter) under long-term low calcium stress
Source: Sci Rep. 2022 Nov 15;12:19552. doi: 10.1038/s41598-022-23844-z (PMC9666473; doi:10.1038/s41598-022-23844-z)

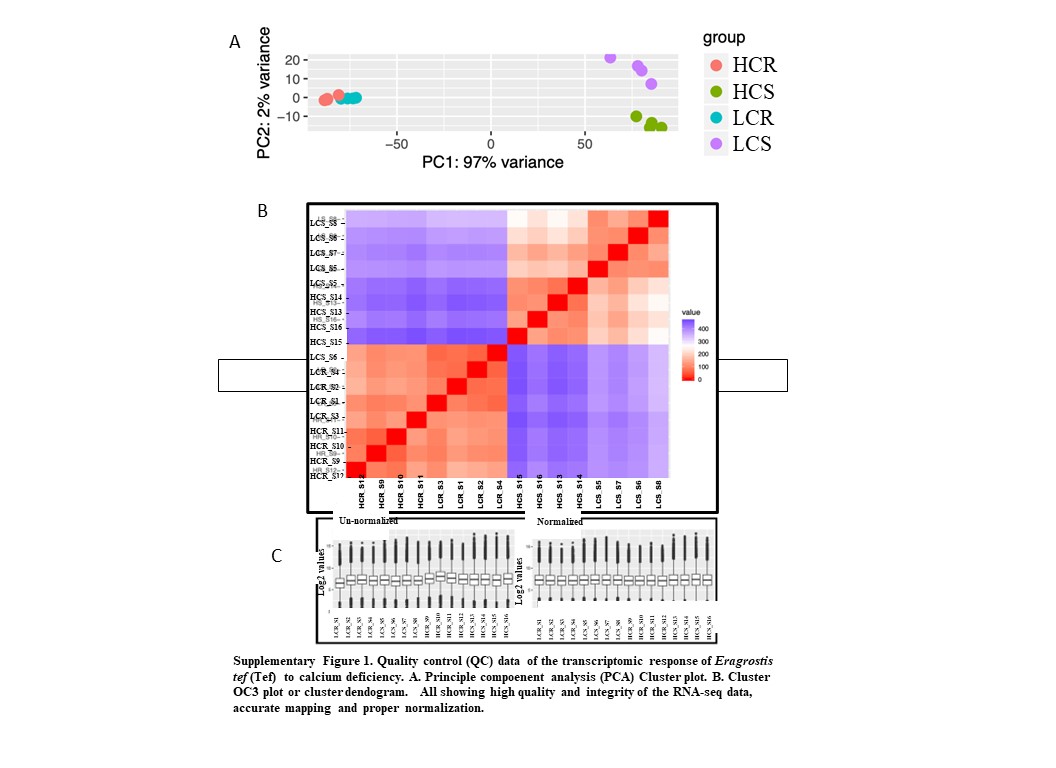

Supplement: Supplementary file 1 — Supplementary Information 1. [file 41598_2022_23844_MOESM1_ESM.jpg]
